# THE ROLE OF GERONTOLOGICAL PSYCHOLOGY IN ACHIEVING THE UN’S SUSTAINABLE DEVELOPMENT GOALS

**DOI:** 10.1093/geroni/igz038.294

**Published:** 2019-11-08

**Authors:** Toni C Antonucci

**Affiliations:** University of Michigan, Ann Arbor, Michigan, United States

## Abstract

The United Nations has identified 17 Sustainable Development Goals (SDGs) designed to improve the health and well-being of the world’s most vulnerable populations. This presentation will review the potential role psychology, in particular, illustrative theories and research, in achieving the SDGs of reducing poverty and achieving gender equality and empowerment. We consider life span (individual) developmental and life course (environmental structure) theories as useful for explaining how poverty and inequality influence the individual and community at one point in time and over time. Further, we use research evidence to illustrate how naturally occurring resources can be garnered to better explain, understand, identify and create successful intervention programs. We emphasize the importance psychology to achieving SDGs and emphasize that the application psychology to changing the behavior and expectations of individuals and societies to achieve sustainable development that contributes to a world that celebrates optimal and sustainable development for all.

